# Bidirectional Association between Self-Reported Hypertension and Gout: The Singapore Chinese Health Study

**DOI:** 10.1371/journal.pone.0141749

**Published:** 2015-10-28

**Authors:** An Pan, Gim Gee Teng, Jian-Min Yuan, Woon-Puay Koh

**Affiliations:** 1 Department of Epidemiology and Biostatistics, Ministry of Education Key Lab of Environment and Health, School of Public Health, Tongji Medical College, Huazhong University of Science and Technology, Wuhan, Hubei, China; 2 Yong Loo Lin School of Medicine, National University of Singapore and National University Health System, Singapore, Republic of Singapore; 3 University Medicine Cluster, Division of Rheumatology, National University Hospital and National University Health System, Singapore, Republic of Singapore; 4 Division of Cancer Control and Population Sciences, University of Pittsburgh Cancer Institute, Pittsburgh, Pennsylvania, United States of America; 5 Department of Epidemiology, University of Pittsburgh Graduate School of Public Health, Pittsburgh, Pennsylvania, United States of America; 6 Duke-NUS Graduate Medical School Singapore, Singapore, Republic of Singapore; 7 Saw Swee Hock School of Public Health, National University of Singapore and National University Health System, Singapore; Shanghai Institute of Hypertension, CHINA

## Abstract

It has been hypothesized that the association between hypertension and gout is bidirectional, however, few studies have examined this in a prospective cohort. We analyzed data from the Singapore Chinese Health Study (SCHS) follow-up I (1999–2004) and II (2006–2010) interviews, when both physician-diagnosed hypertension and gout were self-reported. We included participants with data for both follow-up interviews and who were free of heart disease, stroke and cancer at follow-up I. The analysis of hypertension and risk of gout included 31,137 participants when prevalent gout cases were excluded, while the analysis of gout and risk of hypertension included 20,369 participants when prevalent hypertension cases were excluded. Cox proportional hazards models were used to estimate multivariable-adjusted hazard ratios (HRs) and 95% confidence intervals (CIs). The mean age at follow-up I was 60.1 (SD 7.3) years, and the average follow-up was 6.8 (SD 1.4) years. In the analysis of hypertension and risk of gout, 682 incident cases were identified. Compared to normotensive participants, hypertensive patients had an88% increased risk of developing gout (HR 1.88; 95% CI 1.61–2.21). In the parallel analysis, 5,450 participants reported to have newly diagnosed hypertension during follow-up. Compared to participants without gout, those with gout had an18% increased risk of developing hypertension (HR 1.18; 95% CI 1.02–1.37). The bidirectional association was stronger in normal weight adults compared to overweight/obese individuals (*P*_interaction_ = 0.06 and 0.04, respectively). The hypertension to gout association was stronger in women compared to men (*P*_interaction_ = 0.04), while the gout to hypertension association was evident in women but not in men (*P*_interaction_ = 0.02). In conclusion, our results suggest that the hypertension-gout association is bidirectional in this cohort of Singapore Chinese adults. The potential interactions of the bidirectional association with obesity and sex deserve further investigations.

## Introduction

Hypertension is a leading risk factor for global disease burden, contributing to 7.0% of global disability-adjusted life years [1]. Hypertension is highly prevalent in both developed countries (e.g., about 30% in US adults) [2] and developing countries (e.g., about 30% in Chinese adults) [3]. Meanwhile, gout is a common arthritis caused by deposition of monosodium urate crystals within joints due to hyperuricaemia [4], and is more common in men and elderly populations [5,6]. The prevalence of gout varies dramatically in different populations due to the differences in diagnosis criteria, study population and study design [7]. Studies in US adults have reported a prevalence of self-reported gout to be 4.6% in men and 2.0% in women aged >45 years [8], and a Taiwan survey reported 8.2% in men and 2.3% in women in 2005–2008 [9].

Both hypertension and gout are associated with increased risk of cardiovascular disease morbidity and mortality [10–15]. Therefore, an association between hypertension and gout in middle-aged and elderly individuals deserves careful examination. It has been observed in different populations that hypertension is positively associated with both prevalent and incident gout [16]. Meanwhile, growing evidence has also linked hyperuricaemia with the development of incident hypertension [17]. Although it has been hypothesized that the hypertension-gout relation is bidirectional, few studies have addressed this hypothesis in a prospective setting, particularly in the Asian populations. Therefore, in this study, we aimed to examine the bidirectional association between hypertension and gout in a prospective cohort of middle-aged and older Chinese in Singapore.

## Methods

### Study population

We used data from the Singapore Chinese Health Study (SCHS), a population-based cohort of 63,257 Chinese adults aged 45–74 years at enrolment (1993–1998) [18]. The participants were recruited from two major Chinese dialect groups in Singapore, the Hokkien and Cantonese. Trained interviewers conducted the face-to-face interviews in participants’ homes at recruitment, and obtained the information on demographics, height, weight, tobacco use, physical activity, dietary habits and medical history. Two follow-up interviews were conducted via telephone among surviving participants between 1999 and 2004, and again between 2006 and 2010 to update information on selected lifestyle factors and medical history. The study was approved by the institutional review board of the National University of Singapore, and all participants gave written informed consents.

We used the follow-up I interview (1999–2004) as baseline for our analysis because both gout and hypertension were enquired at this time among 52,322 surviving participants who participated in this interview. During the follow-up II interview (2006–2010), 39,528 participants were re-contacted and information on gout and hypertension was updated.

### Assessment of hypertension and gout

Specifically, at both follow-up interviews, the participants were asked separately if they had been told by doctors that they had hypertension or gout. If the response was “yes”, participants were also asked about the age of first diagnosis. For cases of gout, the interviewers confirmed that the participants had gout but not other arthritis by verifying with the participants that the diagnosis was based on joint pain and swelling attributed to reported hyperuricemia by their physicians. All interviews were tape-recorded and subjected to quality checks.

### Assessment of covariates

At recruitment (1993–1998), participants were asked about their education level, height, weight, tobacco use, physical activity, alcohol intake and medical history. At follow-up I interview (1999–2004), information on body weight, smoking status, alcohol intake and medical history was further updated. Body mass index (BMI) was calculated by weight in kg divided by square of height in meters.

### Statistical analysis

**Fig 1:**
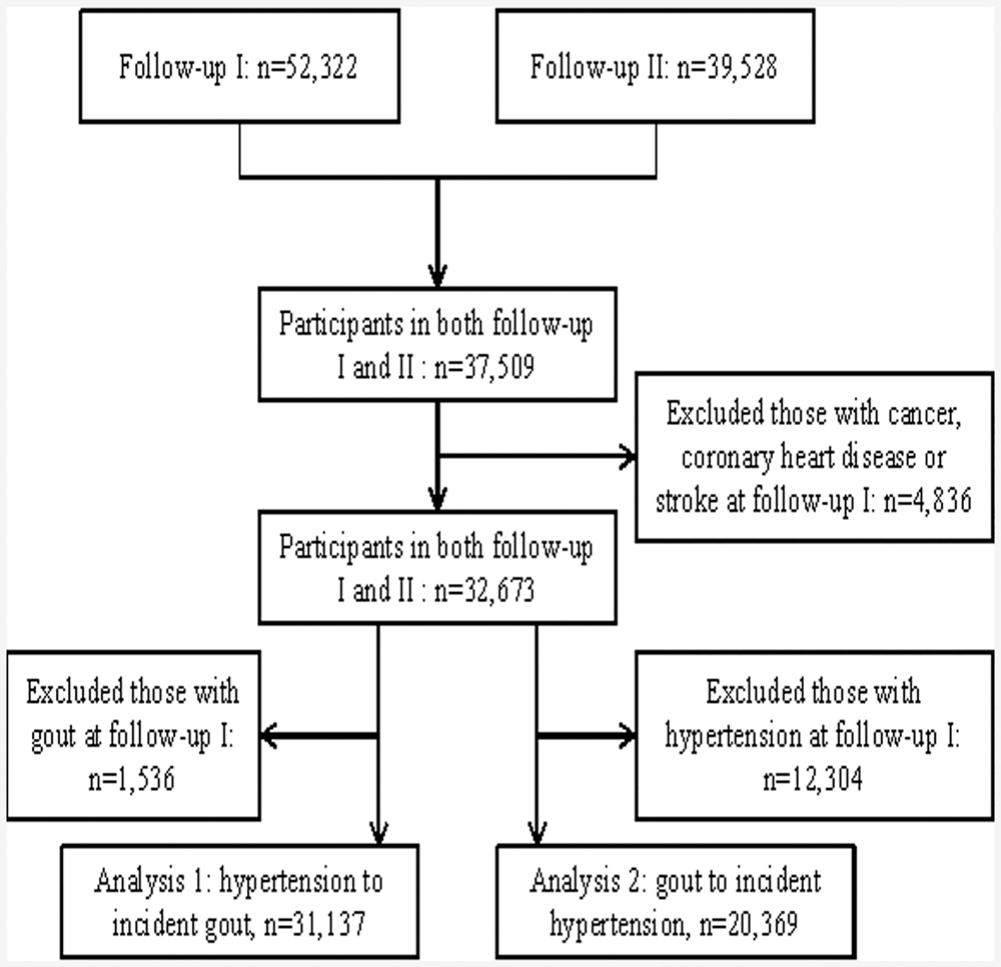
Study flow.

A total of 37,509 individuals participated in both follow-up interviews with complete information on hypertension and gout. We excluded 4,836 participants with history of cancer, coronary heart disease or stroke at follow-up I interview, leaving 32,673 participants for analysis. For the relation of hypertension with incident gout (analysis 1), individuals with history of gout (n = 1,536) were further excluded and the final analysis included 31,137 participants. For the analysis of gout and incident hypertension (analysis 2), individuals with prevalent hypertension (n = 12,304) were further excluded and the final analysis included 20,369 participants. The study flow is shown in Fig 1.

For both analyses, we first compared means or proportions of covariates according to baseline status of hypertension (analysis 1) and gout (analysis 2) at follow-up I interview. For these comparisons, we used χ^2^ tests for categorical variables and t-test for continuous variables. Person-years for each participant were calculated from date of follow-up I interview to the date of reported outcome diagnosis (gout in analysis 1 and hypertension in analysis 2), or the follow-up II interview, whichever occurred first. Cox proportional hazards regression was used to calculate hazard ratio (HR) and its 95% confidence interval (CI), with adjustment for age (years), sex, dialect (Hokkien/Cantonese), year of follow-up I interview (1999–2001, 2002–2004), educational level (none, primary school, secondary school or higher), moderate physical activity level (<0.5, 0.5–3.9, ≥4.0 hours/week), smoking status (never, former, current), alcohol consumption (none, monthly, weekly, daily), BMI (<20.0, 20.0–23.9, 24.0–27.9, ≥28.0 kg/m^2^), and self-reported history of diabetes.

Previous studies have suggested substantial sex differences in prevalence of gout [5.6], and potential sex differences in the relation between hyperuricemia and hypertension [17], thus, we also stratified our analyses by sex. Potential interaction tests were explored with categories for age (<60 and ≥60 years), BMI (<24.0 and ≥240 kg/m^2^), and smoking (never and ever smokers). We also conducted a 2-year lag sensitivity analysis after excluding early-onset cases. All reported *P* values were 2-sided, and statistical analysis was performed with SAS version 9.2 (SAS Institute, Cary, North Carolina, USA).

## Results

The characteristics of the participants at follow-up I interview are shown in Table 1. Compared to normotensive participants, hypertensive patients were older, had higher BMIs and more likely to be current smokers and have diabetes. The differences of other characteristics were small but statistically significant, probably because of large sample size. Compared to individuals without gout, participants with gout at follow-up I visits were younger, had higher BMIs, more likely to be male and former smokers, and to have higher education levels.

**Table 1:** Characteristics of cohort participants at follow-up I interview (1999–2004) in the two analyses: The Singapore Chinese Health Study.

Characteristics	Analysis 1: hypertension to incident gout			Analysis 2: gout to incident hypertension		
	Baseline hypertensive patients	Participants without hypertension	*P* value^a^	Participants with baseline gout	Participants without gout	*P* value^a^
Number of participants	11397 (36.6)	19740 (63.4)		573 (2.8)	19796 (97.2)	
Age, y	61.3 ± 7.3	59.3 ± 7.2	<0.001	58.5 ± 6.6	59.3 ± 7.2	0.005
Body mass index, kg/m^2^	24.1 ± 3.5	22.5 ± 3.3	<0.001	23.9 ± 3.2	22.5 ± 3.3	<0.001
Male	4403 (40.7)	7982 (40.4)	<0.001	346 (60.4)	8010 (40.5)	<0.001
Cantonese Dialect	5748 (50.4)	9712 (49.2)	0.04	279 (48.7)	9746 (49.2)	0.80
Diabetes	2393 (21.0)	1456 (7.4)	<0.001	48 (8.4)	1460 (7.4)	0.37
Education level			<0.001			<0.001
No	3014 (26.5)	4319 (21.9)		65 (11.3)	4326 (21.9)	
Primary school	5119 (44.9)	8855 (44.9)		236 (41.2)	8880 (44.9)	
Secondary school or higher	3264 (28.6)	6566 (33.3)		272 (47.5)	6590 (33.3)	
Smoking status			<0.001			<0.001
Never	8612 (75.6)	14082 (71.3)		365 (63.7)	14120 (71.3)	
Former	1630 (14.3)	2241 (11.4)		108 (18.9)	2248 (11.4)	
Current	1155 (10.1)	3417 (17.3)		100 (17.5)	3428 (17.3)	
Physical activity			<0.001			0.055
<0.5 hours/week	8768 (76.9)	15604 (79.1)		430 (75.0)	15647 (79.0)	
0.5–3.9 hours/week	1674 (14.7)	2735 (13.9)		91 (15.9)	2742 (13.9)	
≥4 hours/week	955 (8.4)	1401 (7.1)		52 (9.1)	1407 (7.1)	
Alcohol intake			<0.001			0.01
Abstainers	10381 (91.1)	17400 (88.2)		482 (84.1)	17448 (88.1)	
Weekly drinkers	743 (6.5)	1743 (8.8)		69 (12.0)	1750 (8.8)	
Daily drinkers	273 (2.4)	597 (3.0)		22 (3.8)	598 (3.0)	

Values are shown in mean ± standard deviation or n (%).

^a^ *P* value was calculated by t-test for continuous variables and χ^2^ test for categorical variables.

### Hypertension and risk of incident gout (analysis 1)

After a mean follow-up of 6.9 (SD 1.3) years, 682 participants reported to have incident gout. Hypertension was associated with an 88% increased risk of developing gout (HR 1.88; 95% CI 1.61–2.21; Table 2). A dose-response relation was observed between duration of hypertension and gout (*P*_trend_<0.001). The association was not materially changed in the 2-year lag analysis (HR 1.85; 95% CI 1.57–2.18).

**Table 2:** Hazard ratios (95% confidence intervals) for risk of gout according to hypertension status: The Singapore Chinese Health Study (1999–2010).

	Cases/person-years	Model 1	Model 2
Baseline (follow-up I) hypertension status			
No	318/136380	1.00 (ref)	1.00 (ref)
Yes	364/77861	2.06 (1.77–2.40)	1.88 (1.61–2.21)
Stratified by sex^a^			
Men			
No	171/54448	1.00 (ref)	1.00 (ref)
Yes	163/29671	1.75 (1.41–2.17)	1.67 (1.33–2.09)
Women			
No	147/81932	1.00 (ref)	1.00 (ref)
Yes	201/48190	2.39 (1.93–2.97)	2.08 (1.66–2.60)
Stratified by BMI category^a^			
Normal weight			
No	180/97749	1.00 (ref)	1.00 (ref)
Yes	160/41705	2.11 (1.70–2.62)	2.11 (1.69–2.64)
Overweight/obesity			
No	138/38631	1.00 (ref)	1.00 (ref)
Yes	204/36156	1.62 (1.30–2.01)	1.66 (1.33–2.07)
Duration of hypertension			
No hypertension	318/136380	1.00 (ref)	1.00 (ref)
0.1–4.9 years	89/25992	1.50 (1.18–1.89)	1.38 (1.09–1.76)
5.0–9.9 years	107/19753	2.39 (1.92–2.98)	2.21 (1.77–2.77)
≥10.0 years	146/24666	2.67 (2.18–3.26)	2.44 (1.98–3.01)
*P* for trend^b^		<0.001	<0.001
2-year lag analysis^c^			
No	300/96918	1.00 (ref)	1.00 (ref)
Yes	337/55094	2.02 (1.73–2.36)	1.85 (1.57–2.18)

Model 1: adjusted for age, sex, dialect, year of interview, and educational level.

Model 2: model 1 plus body mass index, physical activity, smoking status, alcohol use, and history of diabetes at follow-up I.

^a^The *P* for interaction was 0.04 for sex, and 0.06 for BMI category (<24 and ≥24 kg/m^2^).

^b^ *P* for trend was calculated by treating the categorical variable of duration of hypertension as a continuous variable.

^c^45 participants were excluded from the analysis.

We found significant interactions with sex and BMI, but not other variables. The association was slightly stronger in women (HR 2.08; 95% CI 1.66–2.60) compared to men (1.67; 1.33–2.09; *P*_interaction_ = 0.04; Table 2); marginally stronger in normal weight adults (BMI <24 kg/m^2^; 2.11; 1.69–2.64) compared to overweight/obese individuals (BMI ≥24 kg/m^2^; 1.66; 1.33–2.07; *P*_interaction_ = 0.06).

### Gout and risk of incident hypertension (analysis 2)

In the parallel analysis of gout and risk of hypertension, 5,450 participants reported to have new-onset hypertension during a mean follow-up of 6.2 (SD 1.9) years. Compared to participants without gout, those with gout had an 18% increased risk of developing hypertension (HR 1.18; 95% CI 1.02–1.37; Table 3). A dose-response association between duration of gout and risk of hypertension was observed (*P*_trend_ = 0.03; Table 3). The association was not substantially different in the 2-year lag analysis (1.16; 0.99–1.35).

**Table 3:** Hazard ratios (95% confidence intervals) for risk of hypertension according to gout status: The Singapore Chinese Health Study (1999–2010).

	Cases/person-years	Model 1	Model 2
Baseline (follow-up I) gout status			
No	5265/124476	1.00 (ref)	1.00 (ref)
Yes	185/3521	1.28 (1.11–1.49)	1.18 (1.02–1.37)
Stratified by sex^a^			
Men			
No	2049/50003	1.00 (ref)	1.00 (ref)
Yes	120/2058	1.46 (1.21–1.75)	1.31 (1.09–1.58)
Women			
No	3216/74474	1.00 (ref)	1.00 (ref)
Yes	65/1463	1.01 (0.78–1.29)	0.93 (0.72–1.19)
Stratified by BMI category^a^			
Normal weight			
No	3422/90073	1.00 (ref)	1.00 (ref)
Yes	95/1857	1.40 (1.14–1.72)	1.34 (1.09–1.65)
Overweight/obesity			
No	1843/34406	1.00 (ref)	1.00 (ref)
Yes	90/1664	1.01 (0.82–1.25)	1.01 (0.81–1.25)
Duration of gout			
No gout	5265/124476	1.00 (ref)	1.00 (ref)
0.1–4.9 years	90/1767	1.24 (1.01–1.53)	1.14 (0.93–1.41)
≥5.0 years	95/1754	1.33 (1.09–1.63)	1.21 (0.99–1.49)
*P* for trend^b^		0.001	0.03
2-year lag analysis^c^			
No	4922/85227	1.00 (ref)	1.00 (ref)
Yes	169/2391	1.26 (1.08–1.47)	1.16 (0.99–1.35)

Model 1: adjusted for age, sex, dialect, year of interview, and educational level.

Model 2: model 1 plus body mass index, physical activity, smoking status, alcohol use, and history of diabetes at follow-up I.

^a^The *P* for interaction was 0.02 for sex, and 0.04 for BMI category (<24 and ≥24 kg/m^2^).

^b^ *P* for trend was calculated by treating the categorical variable of duration of gout as a continuous variable.

^c^359 participants were excluded from the analysis.

We found significant interactions with sex and BMI, but not other variables. The association was evident in men (HR 1.31; 95% CI 1.09–1.58) but not in women (0.93; 0.72–1.19; *P*_interaction_ = 0.02; Table 3); present in normal weight adults (1.34; 1.09–1.65) but not among overweight/obese individuals (1.01; 0.81–1.25; *P*_interaction_ = 0.04).

## Discussion

The present study, from a large cohort of middle-aged and elderly Chinese in Singapore, is the first prospective study to examine the bidirectional association between hypertension and gout, and adds to the growing evidence that they are closely related to each other. Overall, hypertension was associated with an 88% increased risk of developing gout, while gout was related to an 18% increased risk of incident hypertension. This reciprocal association also depends on sex and baseline obesity status. All the associations were independent of socio-demographic variables and lifestyle factors.

Hypertension is a common comorbidity in patients with gout. It has been estimated that 74% of the gout patients had hypertension in a national survey among US general population [19]. This phenomenon has been observed in other studies as well [20,21], including a large study in Chinese population in Hong Kong [22]. However, the comorbidity of gout and hypertension could be explained by the scenario that gout may be a consequence of having hypertension or as a risk factor for the onset of hypertension. Therefore, the temporal association between the two conditions has attracted a lot of attention in the past several decades [23].

Population-based prospective studies have consistently shown a positive relation between hypertension and risk of developing gout [24–31]. For example, in an early analysis of a 12-year follow-up study from the Health Professional Follow-up Study, Choi et al. [27] found that the multivariable relative risk (RR) was 2.31 (95% CI 1.96–2.72), and this association was independent of diuretic use. In the Atherosclerosis Risk in Communities Study, McAdams-DeMarco et al. [30] found a 2-fold risk of developing gout in hypertensive patients (RR 2.00; 95% CI 1.54–2.61) during 9 years of follow-up, and the association was substantially attenuated after adjustment for serum urate levels. A recent cohort study in Chinese population in Taiwan found a 32%-34% higher risk of gout among hypertensive men and women [31]. Therefore, our findings are broadly consistent with the current literature and demonstrate that hypertension is an independent risk factor for gout development. In our study, we confirmed that the incidence rate standardized to the age structure of the whole cohort was higher in men (3.9 per 1000 person-years) compared to that in women (2.7 per 1000 person-years), which is consistent with the literature that gout is more common in men [5,6]. Interestingly, we found that the relation was slightly stronger in women, which is consistent with a cohort study conducted in a UK general practice database [29]. The Framingham Heart Study also found a slightly stronger association in women compared to that in men, although the interaction was not statistically significant [28], while some other studies did not find the effect modification by sex [30,31]. Thus, whether the association is different in men and women remains unclear. The relatively stronger association in normal weight participants compared to that in overweight/obese individuals suggests that hypertension confers greater risk for gout in lean people. However, a previous study in US population did not found significant interaction with obesity status [30]. The stronger associations in women and normal weight individuals also suggest that the effect of hypertension on risk of gout is relatively greater in lower risk groups.

To the best of our knowledge, our study is the first to report an association between self-reported gout and development of hypertension in a cohort of middle-aged and elderly Chinese. A recent meta-analysis of 18 prospective cohort studies reported that hyperuricemia (by different definitions) was associated with a 41% increased risk of developing hypertension, and each 1 mg/dL increase in uric acid level was associated with a 13% increase in risk [17]. Positive association between uric acid levels and new-onset hypertension has been reported in Chinese populations as well [32–35]. Our study adds further evidence that gout is associated with an increased risk of developing hypertension. We also found significant interaction with sex, and the association was evident in men but not in women. A cross-sectional study in middle-aged Chinese reported that hyperuricemia was associated with higher odds of hypertension in men compared that in women [36]. However, in the above mentioned meta-analysis, stronger association between hyperuricemia and incident hypertension was found in women instead (RR 1.76; 95% CI 1.46–2.05) compared to men (RR 1.38; 95% CI 1.20–1.57). Thus, the sex difference in the association is still inconsistent and deserves further investigation. We also found stronger association in normal weight participants compared to their overweight counterparts, which is consistent with a cohort study in male Japanese workers that serum uric acid was associated with an increased risk of hypertension in leaner men [37].

There are several plausible explanations for the bidirectional association between hypertension and gout. First, hypertension can cause glomerular arteriolar damage and glomerulosclerosis, which then lead to renal insufficiency and decreased renal excretion of urate [38]. The reduced renal blood flow with increased renal and systemic vascular resistance may also contribute to hyperuricemia in hypertensive patients [39]. Furthermore, certain antihypertensive drugs also influence the levels of serum uric acid and thus may contribute to the development of gout. For example, diuretics, β blockers, angiotensin converting enzyme inhibitors, and non-losartan angiotensin II receptor blockers were associated with an increased risk of gout in hypertensive patients [40]. On the other hand, uric acid has a pathogenic role in hypertension [41–43], mediated by several mechanisms such as vascular smooth muscle cell proliferation [44], increased C-reactive protein expression [45], endothelial dysfunction and decreased nitric oxide production [46], and local activation of the renin-angiotensin system [47]. Experimental studies have further shown that a rise in serum uric acid caused by inhibition of uricase in the rats results in systemic hypertension that is preventable by lowering uric acid with either xanthineoxidase inhibitors or uricosuric agents [48–50]. Consistent with the experimental evidence, elevated childhood serum uric acid levels were found to directly correlate with blood pressures in untreated children [51], and also with higher blood pressure levels that persisted into adulthood [52]. Meanwhile, lowering uric acid with allopurinol directly decreased blood pressures in hyperuricaemic adolescents with newly diagnosed hypertension [53], as well as in adults [54,55], suggesting a causal relation between serum uric acid levels and hypertension. Finally, this substantial burden of comorbidity of hypertension and gout may possibly stems from common pathogenesis of the two conditions, such as unhealthy lifestyles, obesity, insulin resistance, and inflammation.

The strength of this study is the large sample with sufficient number of incident gout/hypertension cases identified from a population-based prospective cohort with a relatively long-term follow-up. We have tried to control multiple potential risk factors for gout or hypertension in our analysis. In addition, participants with prevalent history of cancers, coronary heart disease, and stroke were excluded from the analysis since they could be associated with gout or hypertension.

Our study has some limitations. A potential limitation is that both hypertension and gout were self-reported, and we did not collect detailed information on the treatment of the diseases. Previous studies have suggested that anti-hypertensive drugs may contribute to the development of gout, and diuretics, β blockers, and angiotensin converting enzyme inhibitors were associated with an increased risk of gout [40], and those medications have been commonly prescribed to hypertensive patients in Singapore [56]. Therefore, part of the increased risk of developing gout in hypertensive patients might be explained by the effects of drugs. However, some studies have suggested that the association was independent of diuretic use [27,30]. Shortly after Follow-up I, a sub-cohort of the participants (20,013 out of 31,137 participants in analysis 1) responded to supplemental questionnaires with a question about anti-hypertensive drug usage (without information on types of drugs). Among the participants reported to have hypertension, 74.5% of them used medications to control blood pressures; while among those without self-reported hypertension, only 4% used anti-hypertensive drugs (data not shown). This provided a complimentary information about the validity of hypertension in this study, but this was not optimal because the information on medication use was not complete for all participants at all follow-up visits, and was self-reported itself with limited capacity as a gold standard. We did not have information on the serum uric acid levels or renal function, and thus could not test whether the association was due to the relatively higher blood levels of uric acid or higher prevalence of renal impairment in hypertensive patients. A previous study found that the association was substantially attenuated with adjustment for serum urate levels [30]. Furthermore, validation using drug prescription data and medical records for gout and hypertension was not feasible in our study. As for gout, although we had trained our interviewers to further enquire about hyperuricemia in order to increase the accuracy of self-reported gout, misclassification is possible. As with large population-based studies, it is not feasible to require the presence of intra-articular urate crystals or tophus as the gold standard for diagnosis of gout. Although a study in US showed that self-report of physician-diagnosed gout had moderate to good reliability and sensitivity, and the authors have suggested that self-report of physician-diagnosed gout is appropriate for epidemiologic studies [57], the validity of self-reported gout in our study is unknown. Furthermore, the methodology of confirming gout cases by affirmative answer to the question “Have you been told by a doctor to have gout?” has been commonly used in other population-based large cohort studies [11,58]. Surveillance bias due to disease diagnosis is also a possible explanation for our findings, since people with hypertension or gout may be more likely to see the doctors and get their blood pressures or serum uric acid levels measured. We have applied the 2-year lag analysis and the results remained unchanged; however, we do not have information on frequency of general practitioner visits and could not fully account for this bias.

In conclusion, our prospective cohort study provides evidence of a bidirectional association between hypertension and gout, and the association may be modified by sex and obesity status. Our study has been limited by the self-reported measures of hypertension and gout, thus, more studies with validated assessments of both hypertension and gout are warranted. Future studies are also needed to confirm our findings in different populations and to investigate the potential mechanisms. If they can be replicated, our findings have significant public health and clinical importance, because both conditions are common in Asia and globally, and that they are major risk factors for cardiovascular disease and premature death.
